# Supplementary figures and images for: Structural insights into the RNA interaction with Yam bean Mosaic virus (coat protein) from Pachyrhizus erosus using bioinformatics approach
Source: PLoS One. 2022 Jul 22;17(7):e0270534. doi: 10.1371/journal.pone.0270534 (PMC9307209; doi:10.1371/journal.pone.0270534)

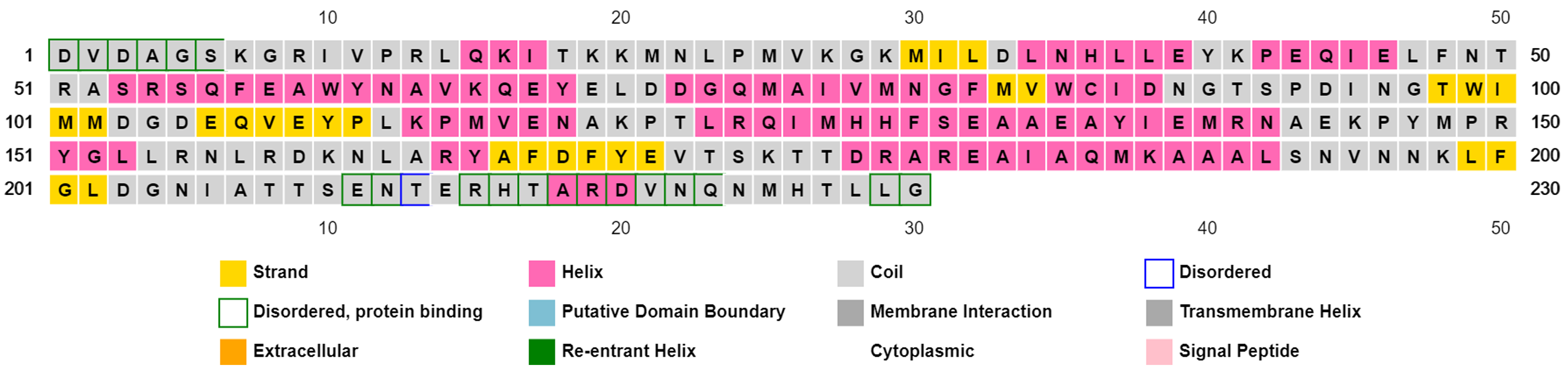

Supplement: S1 Fig — The figure represents the secondary structure map where query sequence and coloured residues are presented as per the annotations made by each analysis methods. Map shows helical residues that are coloured in pink, b-strand residues coloured in yellow, putative domain boundaries are indicated in blue, and green bordered box represents disordered protein binding. (TIF) [file pone.0270534.s001.tif]

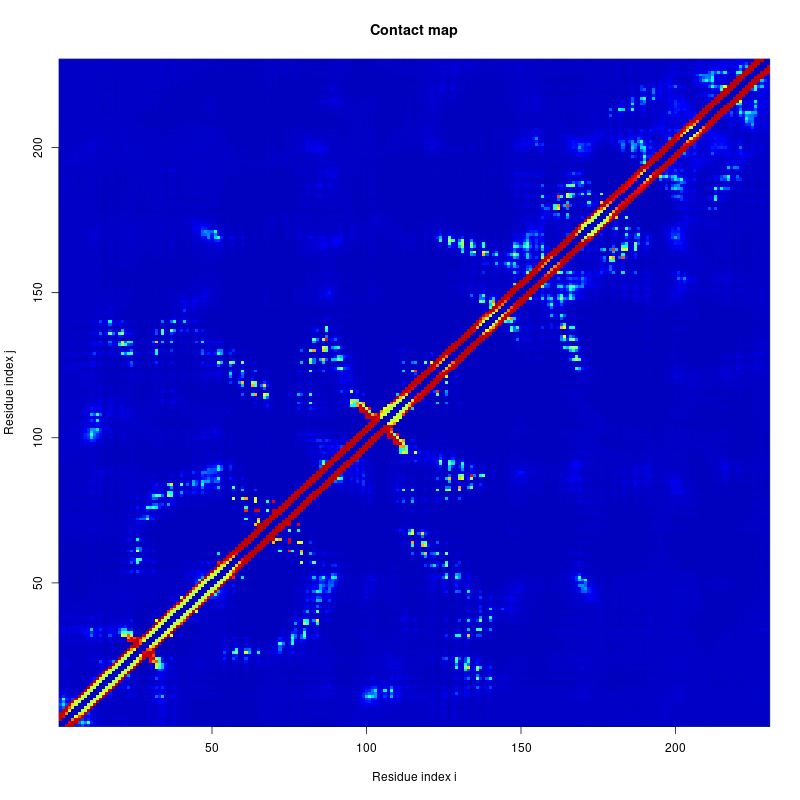

Supplement: S2 Fig — The figure shows the contact map of YBMV protein model. The map shows a matrix with contacts with the color scale which represents the relationship between the amino acids in the particular map. (TIF) [file pone.0270534.s002.tif]

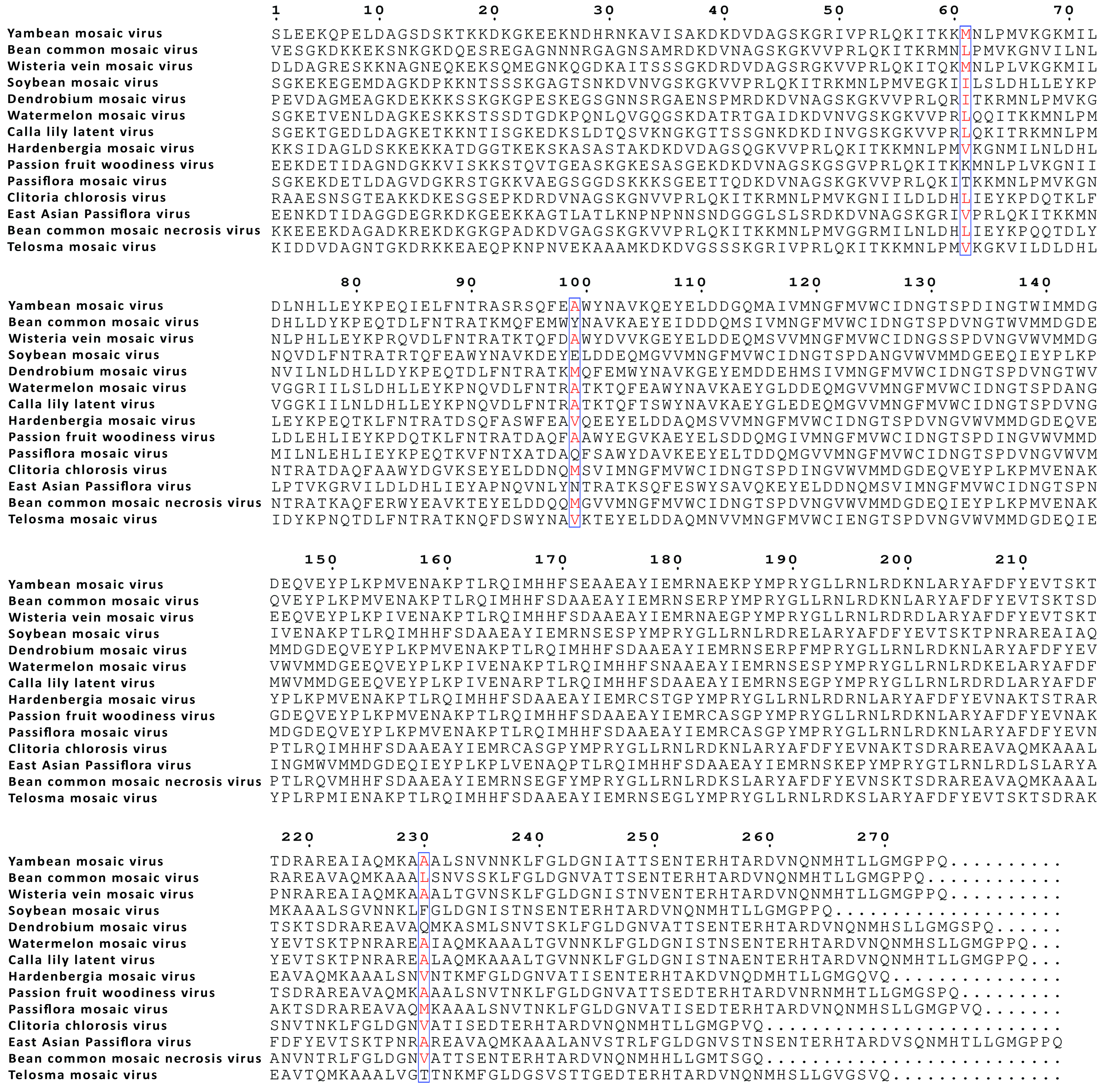

Supplement: S3 Fig — Multiple sequence alignment of YMBV_CP (coat protein) sequence with other CP viruses from the potyvirus family. The image is generated in Multalin software. (TIF) [file pone.0270534.s003.tif]

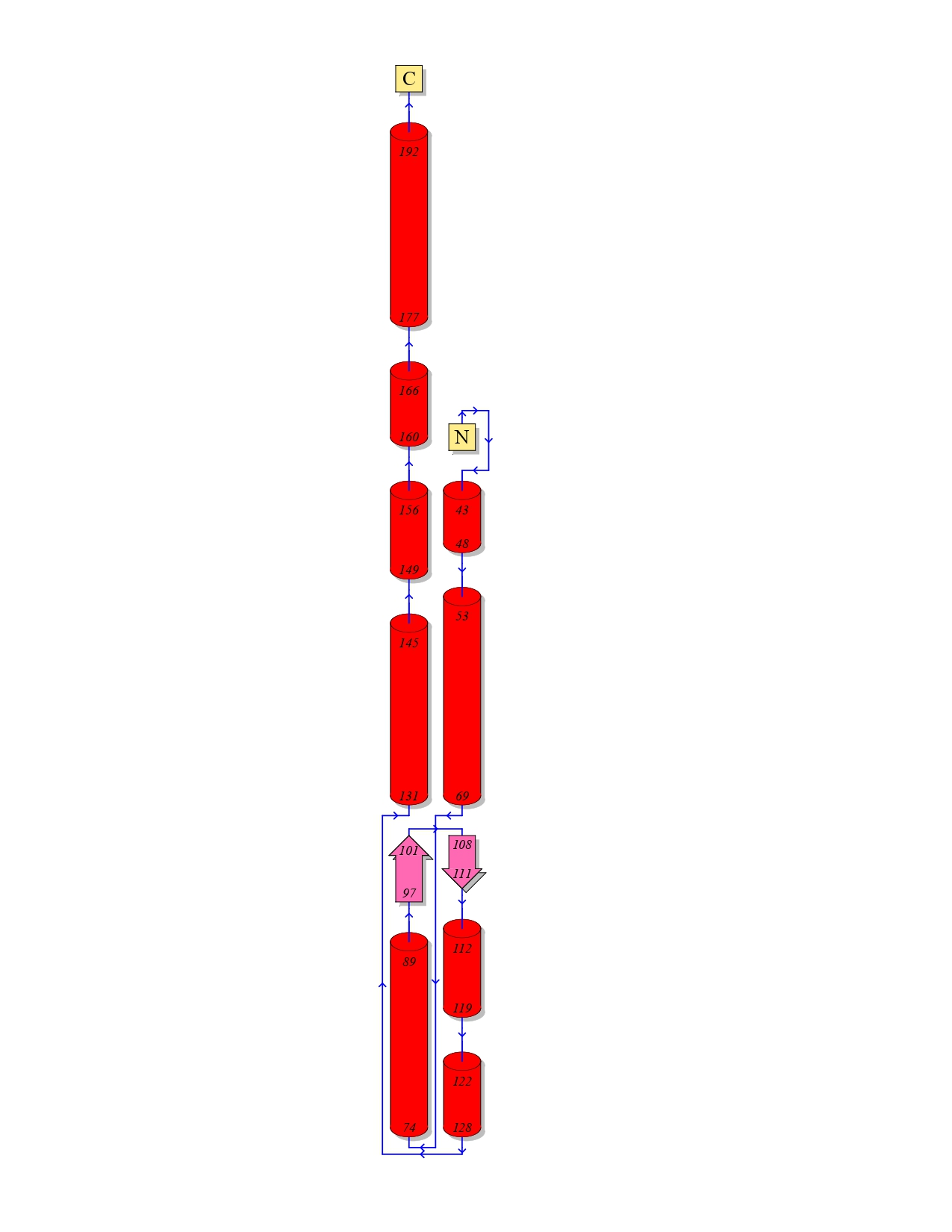

Supplement: S4 Fig — Protein topology map, constructed using Procheck, with beta-strands as a pink-coloured arrow and the alpha-helices as red cylinders. (TIF) [file pone.0270534.s004.tif]
