# Supplementary material for: Structural insights into the RNA interaction with Yam bean Mosaic virus (coat protein) from Pachyrhizus erosus using bioinformatics approach
Source: PLoS One. 2022 Jul 22;17(7):e0270534. doi: 10.1371/journal.pone.0270534 (PMC9307209; doi:10.1371/journal.pone.0270534)
Supplement: S1 Table — The full-length sequence acc. no. of the coat protein of potyvirus and identification of protein domain using the conserved domain search. (DOC) [file pone.0270534.s005.doc]

**S1 Table**. The full-length sequence acc. no. of the coat protein of potyvirus and identification of protein domain using the conserved domain search

| **Sl.No** | **Potyvirus** | **Acronym** | **NCBI Accession** | **Interval** | **Protein Length** | **E-value** |
| --- | --- | --- | --- | --- | --- | --- |
| 1. | Bean common mosaic virus | BCMV | NP_734122 | 53-282 | 230 aa | 5.55e-102 |
| 2. | Bean yellow mosaic virus | BYMV | NP_734182 | 40-272 | 233 aa | 1.53e-109 |
| 3. | Beet mosaic virus | BMV | NP_954628 | 48-274 | 277 aa | 9.55e-102 |
| 4. | Chilli veinal mottle virus | CVMV | NP_982344 | 53-285 | 233 aa | 3.76e-117 |
| 5. | Cocksfoot streak virus | CSV | NP_734398 | 49-281 | 233 aa | 5.34e-94 |
| 6. | Cowpea aphid-borne mosaic virus | CABMV | NP_734388 | 41-270 | 230 aa | 8.46e-101 |
| 7. | Dasheen mosaic virus | DMV | AJ298033 | 79-310 | 232 aa | 1.06e-110 |
| 8. | Japanese yam mosaic virus | JYMV | BAA36278 | 2896-3128 | 233 aa | 1.63e-107 |
| 9. | Johnson grass mosaic virus | JGMV | Z26920 | 70-302 | 233 aa | 1.22e-102 |
| 10. | Leek yellow stripe potyvirus | LYS | AJ307057 | 53-285 | 233 aa | 4.75e-100 |
| 11. | Lily mottle virus | LMV | AJ564636 | 41-271 | 231 aa | 3.96e-107 |
| 12. | Maize dwarf mosaic virus | MDMV | AJ001691 | 59-291 | 233 aa | 5.43e-98 |
| 13. | Onion yellow dwarf virus | OYDV | AJ510223 | 24-256 | 233 aa | 7.41e-105 |
| 14. | Papaya ringspot virus | PRV | NP_734242.1 | 54-286 | 233 aa | 1.93e-111 |
| 15. | Peanut mottle virus | PMV | AF023848 | 43-276 | 234 aa | 3.43e-104 |
| 16. | Potato virus V | PVV | AJ243766 | 38-270 | 233 aa | 1.23e-116 |
| 17. | Soybean mosaic virus | SMV | NP_734200 | 31-260 | 230 aa | 3.15e-105 |
| 18. | Yam bean mosaic virus | YBMV | YP_004940328 | 41-270 | 230 aa | 6.85e-102 |
| 19. | Yam mosaic virus | YMV | YP_022760 | 68-300 | 233 aa | 1.87e-100 |
| 20. | Zucchini yellow mosaic virus | ZYMV | NP_734192 | 45-276 | 232 aa | 6.06e-105 |
| 21. | Turnip mosaic virus | TMV | BBO25397 | 2930-3162 | 233 aa | 6.21e-107 |
| 22. | Watermelon mosaic virus | WMV | NC_006262 | 47-276 | 230 aa | 4.17e-108 |
| 23. | Wild potato mosaic virus | WPMV | AJ437279 | 38-270 | 233 aa | 5.90e-117 |
| 24. | Sugarcane mosaic virus | ScMV | NP_734142 | 78-310 | 233 aa | 6.59e-100 |
| 25. | Sweet potato feathery mottle virus] | SPFMV | NP_734318 | 80-312 | 23aa | 1.27e-102 |
| 26. | Sorghum mosaic virus | ShMV | NP_734092 | 86-318 | 233 aa | 2.39e-100 |
