# Supplementary material for: Structural insights into the RNA interaction with Yam bean Mosaic virus (coat protein) from Pachyrhizus erosus using bioinformatics approach
Source: PLoS One. 2022 Jul 22;17(7):e0270534. doi: 10.1371/journal.pone.0270534 (PMC9307209; doi:10.1371/journal.pone.0270534)
Supplement: Attachment — Submitted filename: Response to Reviewer.doc [file pone.0270534.s006.doc]

**Manuscript Number: PONE-D-22-04164**

**Point-to-point response to the Reviewers' comments**

We thank the editor for the positive evaluation of our paper, and the two reviewers for recognizing the merit of our work and for providing very valuable suggestions. As detailed below, we have addressed the Reviewers’ comments carefully and revised the manuscript accordingly. To facilitate the reviewing process, we have copied the Reviewers’ original comments, which are shown in black (italic font) and our responses are then shown in blue.

**Reviewer #1**

**Comments 1**

*Authors have not provided the name of the template used for model generation in the abstract.*

**Authors Response**

The template used for the model generation is 5ODV. We have added the template name in the abstract section of the Ms.

**Comments 2**

*Authors have suggested to rewrite the results and conclusion part of the abstract based on findings. In addition, the authors have advised adding a brief description of methodology in the abstract.*

**Authors Response**

As per the suggestion, the results, conclusion and methodology part has been revised accordingly in the abstract section of the Ms.

**Comments 3**

*Objective of the study should be clear at the end of the introduction section.*

**Authors Response**

Thank you for your response. We have modified the introduction section as desired by you.

**Comments 4**

*Authors have been advised to correct the spelling mistakes throughout the manuscript. For example, “formatted”.*

**Authors Response**

We have corrected our spelling mistakes throughout the manuscript.

**Comments 5**

*In the Introduction section the author should refer to the research paper and comment on recent in-silico techniques. It will be good information for the readers. I would like to recommend several papers, among many others, providing further explanation on this topic: PMID: 34717229 PMID: 33465692 PMID: 33749525 PMID: 29380245 PMID: 31933177 PMID: 32353478 PMID: 33993976*

**Authors Response**

Thank you for your valuable comments. These publications are highly useful regarding *in silico* techniques and we have also cited the same in the Ms accordingly.

**Comments 6**

*Provide citations for all the PDB IDs mentioned in the manuscript.*

**Authors Response**

We have cited all PDB IDs in the Manuscript.

**Comments 7**

*Authors should include studies with RNA on the predicted binding site. This could be done by docking and MD simulations.*

**Authors Response**

Thank you for your suggestion. We have performed an MD simulation of 50ns using Desmond with Optimized Potentials for Liquid Simulations (OPLS) 2005 force field.

**Comments 8**

*MD simulations should be carried out to identify key structural changes and residues involved in protein-RNA interactions.*

**Authors Response**

As suggested, we performed an MD simulation to analyze the key structural changes and residues involved in protein-RNA interaction in the Ms.

**Comments 9**

*Clearly mention the aim and objective of the study in the last paragraph of the introduction section.*

**Authors Response**

The aim and objective of the study have been rectified and explained properly in the introduction section of the Ms.

**Comments 10**

*Including limitations of the study in the discussion and conclusions section.*

**Authors Response**

We have modified the discussion and conclusion section as per your suggestion.

**Comments 11**

*Authors have not provided references regarding servers used in the studies.*

**Authors Response**

Thank you for your recommendation. We have cited some references in the Manuscript.

**Comments 12**

*Figure 6 is not visible authors have advised redrawing the figure.*

**Authors Response**

We have revised figure 6 as per your suggestion.

**Comments 13**

*Authors have advised to include more rigorous analysis and validate the findings by experimental/computational analyses.*

**Authors Response**

We have performed a molecular dynamics simulation to validate more findings in computational bioinformatics techniques.

**Comments 14**

*Authors have not compared their findings with already present literature based on similar studies.*

**Authors Response**

We have revised the Manuscript with recent literature studies.

**Comments 15**

*Overall, the manuscript is written in a very confusing and poor way. The writing needs substantial improvement. Need to edit the English language through a native speaker or by other means.*

**Authors Response**

We apologize for the inconvenience. We have revised the manuscript accordingly. We have used Grammarly software and corrected the spelling and grammatical errors present in the manuscript and rewritten the portion that was confusing for the reviewer.

**Reviewer #2**

**Comments 1**

*There is no experimental evidence for their finding. I recommend the authors plan a wet lab experiment to demonstrate the role of those amino acids in RNA binding. For instance, make mutation of those amino acid residues by SDM and test the ability of mutant proteins to bind RNA by EMSA, fluorescent spectroscopy, etc.*

**Authors Response**

Thank you very much for your recommendation. We agree with the reviewer on the above remarks that, the findings from the computational study should be validated through an experimental approach. We are a small group of researchers working in computational biology without the support of a wet-lab experimental setup, therefore, we apologize that the suggested experiments by the esteemed reviewer could not be performed at present MS. However, we strongly believe that our computational findings will be helpful for the researchers working in this area to plan and undertake such studies involving site-directed mutagenesis, EMSA and fluorescent spectroscopy studies which will unravel the binding of RNA to the protein. To additionally, support our study, we have performed all-atoms MD simulation for 50 ns to analyze the structural changes and residues involved in protein-RNA interaction and stability of the complex through RMSD, RMSF, Rg and H-bond analysis (Please see in Fig. 7 of the Ms).

**Comment 2**

*The manuscript is not written well.*

**Authors Response**

Thank you for your suggestion. We have revised the manuscript rigorously and rectified the grammatical mistakes throughout the manuscript.

**Specific comments:**

**Comments 3**

*Page 3, Line 15: “Chenopodium amanticolor, Nicotiana megalosiphon and Nicotiana benthamiana”. Scientific name should be italicized.*

**Authors Response**

We have corrected the scientific name in italics.

**Comments 4**

*Page 3, Line 80: “Sorensen” since there are more than one author in this study, you should mention as Sorensen et al.,*

**Authors Response**

We have revised the Sorensen as Sorensen et al., in the Ms.

**Comments 5**

*Page 3, Line 86: Similar to previous comment. Change “Damayanti” to ““Damayanti et al”*

**Authors Response**

We have corrected the “Damayanti” to “Damayanti et al” in the introduction section.

**Comments 6**

*Page 4, Line 88: “Afterward in……”. Not scientific. Rephrase this sentence.*

**Authors Response**

As per the reviewer's suggestion, we have rewritten the above line.
