# Supplementary material for: Structural insights into the RNA interaction with Yam bean Mosaic virus (coat protein) from Pachyrhizus erosus using bioinformatics approach
Source: PLoS One. 2022 Jul 22;17(7):e0270534. doi: 10.1371/journal.pone.0270534 (PMC9307209; doi:10.1371/journal.pone.0270534)
Supplement: Attachment — Submitted filename: Response to Reviewer.doc [file pone.0270534.s007.doc]

**Manuscript Number: PONE-D-22-04164R2**

**Point-to-point response to the Reviewers' comments**

We thank the editor for the positive evaluation of our paper, and the two reviewers for recognizing the merit of our work and for providing very valuable suggestions. As detailed below, we have addressed the Reviewers’ comments carefully and revised the manuscript accordingly. To facilitate the reviewing process, we have copied the Reviewers’ original comments, which are shown in black (italic font) and our responses are then shown in blue.

**Reviewer #1**

**Comments**

*The authors have responded to all the concerns meticulously and improved the manuscript accordingly.The revised draft is improved significantly. I do not have further comments.*

**Authors Response**

Thank you for your possetive response.

**Reviewer #2**

**Comments 1**

*Generate simulation of mutant protein structure*

**Authors Response**

Thank you very much for your recommendation. As per your suggestion, we have generated the mutant structure of R124A which plays important role in the interaction of YBMV_CP with RNA and maintained stable connection throughout the simulation. All the simulation analysis have been included in the MS.

**Comment 2**

*Determining the wildtype and mutant protein affinity for RNA by Haddock score calculation.*

**Authors Response**

Thank you for your suggestion. We have revised the manuscript accordingly. The HADDOCK score for the wild type and mutant type have been presented in the manuscript.

**Comments 3**

*Identify the pocket that binds to RNA, and the amino acid residues involved in the interaction in both wild-type and mutant protein.*

**Authors Response**

We have identified the pocket that binds to RNA, and the amino acid residues involved in the interaction in both wild-type and mutant type.

**Comments 4**

*To perform these experiments, I suggest authors to cite and follow the following publications.*

*Gnanasekaran, P., Gupta, N., Ponnusamy, K., and Chakraborty, S. (2021). Betasatellite encoded βC1 protein exhibitsnovel ATP hydrolysis activity that influences its DNA-binding activity and viral pathogenesis. Journal of Virology, 95(20): e00475-21.*

*Gnanasekaran, P., Ponnusamy, K., and Chakraborty, S. (2019) A geminivirus betasatellite encoded βC1 proteininteracts with PsbP and subverts PsbP-mediated antiviral defense in plants. Molecular Plant Pathology, 20(7): 943-960.*

**Authors Response**

Thank you for your suggestion. We have cited the two references in the Manuscript.
